# BDNF-TrkB Signaling Engages a Src Family Kinase-Pannexin 1 Pathway During the Onset of Sustained Mechanical Hyperalgesia

**DOI:** 10.3390/ijms27146510

**Published:** 2026-07-22

**Authors:** Jonathan Aránguiz Barrera, Ana María Moreira-Banuth, Katherine Zepeda-Morales, María Triolo, Nicolas I. Oneto, David Bravo, Juan Pablo Huidobro-Toro, María Verónica Donoso, Rodrigo Noseda, Teresa Pelissier, Alejandro Hernández, Luis Constandil, Jeffri S. Retamal

**Affiliations:** 1 Laboratory of Neurobiology, Faculty of Chemistry and Biology, University of Santiago of Chile, Ave. Alameda Libertador Bernardo O’Higgins 3363, Santiago 9170022, Chile; jonathan.aranguiz@uv.cl (J.A.B.); ana.moreira@usach.cl (A.M.M.-B.); katherine.zepeda@usach.cl (K.Z.-M.); maria.triolo@usach.cl (M.T.); nicolas.oneto@usach.cl (N.I.O.); david@pannextherapeutics.com (D.B.); juan.garcia-huidobro@usach.cl (J.P.H.-T.); maria.donoso.g@usach.cl (M.V.D.); alejandro.hernandez@usach.cl (A.H.); 2 Kinesiology School, Faculty of Medicine, University of Valparaíso, Valparaíso 2340000, Chile; 3 Department of Anesthesia, Critical Care and Pain Medicine, Beth Israel Deaconess Medical Center, Harvard Medical School, Boston, MA 02215-5400, USA; rnoseda@bidmc.harvard.edu; 4 Faculty of Medicine, Clínica Alemana University del Desarrollo, Santiago de Chile 7610315, Chile; tpelissier@udd.cl

**Keywords:** tropomyosin receptor kinase B, Src-family kinases protein, pannexin 1, mechanical hyperalgesia, pain onset, spinal cord

## Abstract

Brain-derived neurotrophic factor (BDNF) is a key mediator of central sensitization and chronic pain through activation of TrkB receptors. Although the Pannexin 1 (Panx1) channel has been implicated in chronic pain, its involvement in BDNF-TrkB signaling remains unclear. Here, we investigated the functional relationship between TrkB activation and Panx1 using a BDNF-induced pain model in rats. Animals received a single intrathecal administration of BDNF, and mechanical nociception was assessed using the Randall–Selitto test. Behavioral analyses were combined with pharmacological interventions, Western blotting, confocal microscopy, YOPRO-1 uptake assays, and ATP quantification in spinal cord tissue. Intrathecal BDNF induced a robust mechanical hyperalgesia that persisted for up to 10 days. Blockade of Panx1 with 10Panx significantly attenuated BDNF-induced hyperalgesia. BDNF increased Src416 phosphorylation, Panx1 phosphorylation, and YOPRO-1 uptake in dorsal horn neurons, indicating enhanced channel activation. These effects were prevented by the TrkB antagonist ANA12, demonstrating that Panx1 acts downstream of BDNF-TrkB signaling. Inhibition of Src-family kinases with PP2 reduced both hyperalgesia and Panx1 activation, supporting a TrkB-Src-Panx1 signaling cascade. Furthermore, BDNF enhanced ATP release from spinal cord slices, an effect abolished by ANA12, PP2, and 10Panx. Together, these findings identify Panx1 as a downstream effector engaged by BDNF-TrkB signaling during the onset of mechanical hyperalgesia. While persistent TrkB activation appears to be required for the prolonged nociceptive state, the Src family kinase-Panx1 pathway contributes primarily to the early phase of BDNF-induced sensitization.

## 1. Introduction

Chronic pain is a debilitating condition characterized by persistent or recurrent pain that extends beyond the normal period of tissue healing. It affects hundreds of millions of individuals worldwide and represents a major public health burden due to its high prevalence, substantial socioeconomic impact, and limited response to currently available therapies [1,2,3]. Despite significant advances in pain research, the molecular and cellular mechanisms that drive the transition from acute nociceptive responses to persistent pain states remain incompletely understood.

Central sensitization is widely recognized as a key process underlying the development and maintenance of chronic pain [4,5]. This phenomenon involves activity-dependent changes in spinal cord neurons that increase neuronal excitability and amplify nociceptive transmission. Several ion channels, neurotransmitter receptors, and intracellular signaling pathways have been implicated in central sensitization, although the functional interactions among these mechanisms remain incompletely defined [6,7,8,9]. Among the molecular mediators associated with central sensitization, brain-derived neurotrophic factor (BDNF) has emerged as a critical regulator of pain-related neuronal plasticity. BDNF modulates neuronal excitability, synaptic transmission, and gene expression through activation of its high-affinity receptor, tropomyosin receptor kinase B (TrkB) [10,11,12]. Following nerve injury, spinal BDNF levels increase, contributing to the development of chronic pain by enhancing excitatory neurotransmission and promoting long-lasting changes in dorsal horn neuronal function [13,14]. Consistent with this role, intrathecal administration of BDNF produces prolonged nociceptive hypersensitivity and central sensitization in rodents [15,16,17]. Activation of TrkB receptors initiates several downstream signaling cascades, including the recruitment of Src family kinases [10,18,19,20]. Src-dependent phosphorylation is known to regulate the activity of multiple proteins involved in nociceptive processing, including NMDA receptors and the Pannexin 1 (Panx1) channel [21,22,23,24]. Panx1 forms large-pore membrane channels that release ATP and other signaling molecules, thereby contributing to neuron-glia communication and pain-related plasticity. Increasing evidence indicates that Panx1 plays a critical role in the initiation and maintenance of chronic pain, as pharmacological inhibition or genetic deletion of Panx1 reduces nociceptive sensitization and ATP-dependent signaling in several experimental pain models [23,25,26,27]. Although both BDNF-TrkB signaling and Panx1 channel activity have independently been implicated in several experimental models of persistent pain, whether Panx1 functions as a downstream effector of TrkB activation remains unknown. Given that TrkB signaling activates Src-family kinases and that Src-mediated phosphorylation can regulate Panx1 channel activity, we hypothesized that BDNF-induced TrkB activation promotes Src-dependent Panx1 opening in spinal dorsal horn neurons, leading to ATP release and sustained nociceptive sensitization. Thus, understanding the intricate interplay between BDNF-TrkB, Src-family kinases, and Panx1 signaling pathways in pain processing may unveil novel therapeutic targets and strategies for managing chronic pain. To test this hypothesis, we combined behavioral, pharmacological, biochemical, and imaging approaches in a rat model of BDNF-induced pain to investigate the functional relationship among TrkB, Src-family kinases, and Panx1 signaling in the spinal cord.

## 2. Results

### 2.1. Intrathecal BDNF Induces Sustained TrkB-Dependent Mechanical Hyperalgesia in Wild-Type Rats

**Figure 1:**
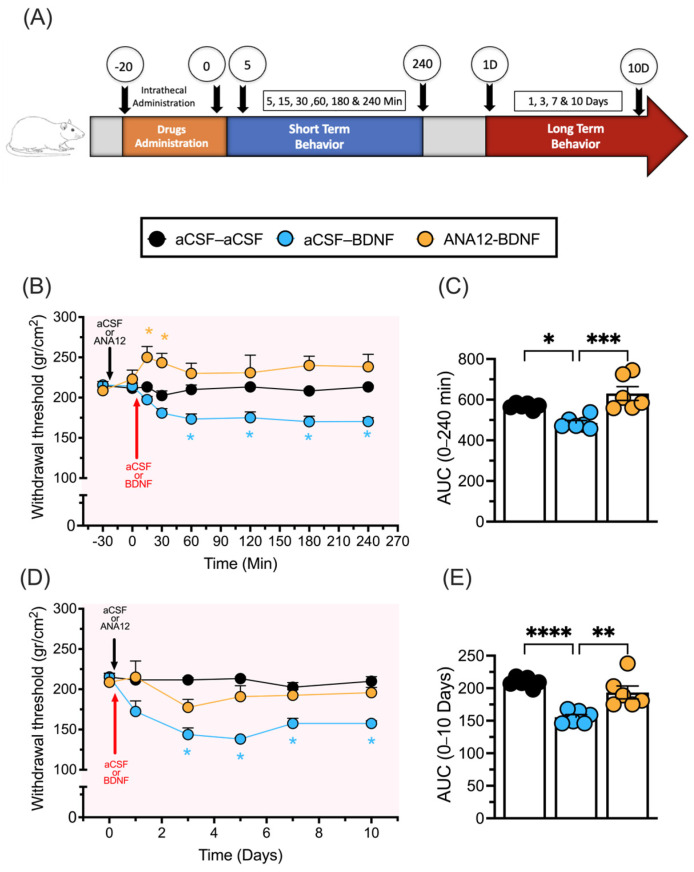
BDNF-TrkB signaling induces sustained mechanical hyperalgesia in wild-type rats. Effect of intrathecal (i.t.) administration of BDNF on short- and long-term nociceptive responses in wild-type rats. (**A**) Experimental timeline showing pretreatment, treatment administration, and assessment of mechanical hypersensitivity using the Randall–Selitto test. (**B**) Short-term (minutes) and (**D**) long-term (days) time-course effects of intrathecal administration of aCSF, BDNF (100 ng), and ANA12/BDNF (100 nM/100 ng). Integrated responses are shown as area under the curve (AUC) for (**C**) short-term (0–240 min) and (**E**) long-term (0–10 days) responses. Data are expressed as mean ± SD (*n* = 6 animals per group). Changes over time within each group were analyzed using the Friedman test, followed by Dunn’s multiple comparisons test. Comparisons between groups at individual time points were performed using the Kruskal–Wallis test, followed by Dunn’s multiple comparisons test. The asterisks shown in the time-course panels indicate significant differences between experimental groups at the corresponding time points. AUC data were analyzed using the Kruskal–Wallis test followed by Dunn’s multiple-comparisons test. * *p* < 0.05, ** *p* < 0.01, *** *p* < 0.001, **** *p* < 0.0001.

To establish the experimental model and determine whether BDNF-induced nociceptive sensitization depends on TrkB signaling, mechanical nociceptive thresholds were evaluated following a single intrathecal administration of BDNF (100 ng/10 µL) in wild-type rats (Figure 1A). Consistent with previous reports demonstrating the pronociceptive actions of BDNF in the spinal cord [11,15,16], intrathecal BDNF produced a rapid reduction in hind paw withdrawal thresholds compared with vehicle-treated animals, indicating the development of mechanical hyperalgesia (Figure 1B). The reduction in nociceptive thresholds was evident within minutes after BDNF administration and persisted throughout the acute observation period (0–240 min). Analysis of the area under the curve (AUC) confirmed a significant decrease in mechanical thresholds during this acute phase compared with vehicle-treated animals (Figure 1C). Importantly, the hyperalgesic response was not transient. Animals receiving BDNF continued to exhibit reduced withdrawal thresholds during the long-term observation period, and AUC analysis demonstrated that the effect persisted for at least 10 days, the longest time point evaluated in this study (Figure 1D,E). These findings confirm that a single intrathecal administration of BDNF is sufficient to induce a prolonged state of mechanical hypersensitivity and support the use of this model to investigate molecular mechanisms underlying sustained nociceptive sensitization.

To determine whether the hyperalgesic effects of BDNF were mediated by TrkB receptor activation, animals were pretreated with the selective TrkB antagonist ANA12 prior to BDNF administration. ANA12 markedly attenuated the reduction in withdrawal thresholds induced by BDNF during both the acute and long-term phases of the response (Figure 1B,D). Notably, ANA12-treated animals exhibited nociceptive thresholds comparable to those of vehicle-treated controls throughout the experimental period. Consistent with these observations, AUC analysis revealed a significant prevention of BDNF-induced hyperalgesia in ANA12-treated animals in both the short and long-term paradigms (Figure 1C,E). Together, these results demonstrate that intrathecal BDNF induces robust and persistent mechanical hyperalgesia in wild-type rats and establish that the development and maintenance of this nociceptive state depend on TrkB receptor activation.

### 2.2. Pharmacological Blockade of Panx1 Attenuates BDNF-Induced Mechanical Hyperalgesia

**Figure 2:**
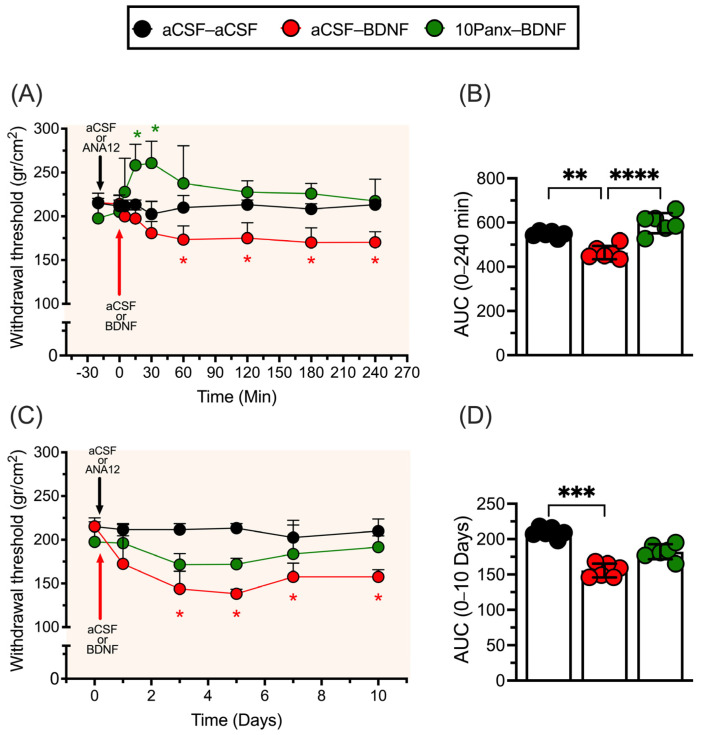
BDNF-induced sustained mechanical hyperalgesia requires Panx1 channel activity in wild-type rats. Effect of intrathecal (i.t.) administration of aCSF, BDNF, and 10Panx on early and late nociceptive responses assessed using the Randall–Selitto test in wild-type rats. (**A**) Short-term (minutes) and (**C**) long-term (days) time-course effects of intrathecal administration of aCSF, BDNF (100 ng), and 10Panx/BDNF (300 µM/100 ng). Integrated responses are shown as area under the curve (AUC) for (**B**) short-term (0–240 min) and (**D**) long-term (0–10 days) responses. Data are expressed as mean ± SD (*n* = 6 animals per group). Changes over time within each group were analyzed using the Friedman test, followed by Dunn’s multiple comparisons test. Comparisons between groups at individual time points were performed using the Kruskal–Wallis test, followed by Dunn’s multiple comparisons test. The asterisks shown in the time-course panels indicate significant differences between experimental groups at the corresponding time points. AUC data were analyzed using the Kruskal–Wallis test, followed by Dunn’s multiple comparisons test. * *p* < 0.05, ** *p* < 0.01, *** *p* < 0.001, **** *p* < 0.0001.

Having established that BDNF-induced hyperalgesia depends on TrkB receptor activation, we next investigated whether Panx1 channels contribute to the nociceptive response induced by BDNF. To this end, wild-type rats received intrathecal administration of the Panx1 inhibitory peptide 10Panx, a pharmacological inhibitor previously shown to reduce pain-related signaling and nociceptive sensitization [24,25,28], prior to BDNF treatment, and mechanical sensitivity was monitored over time (Figure 2A). Consistent with the results presented in Figure 1, intrathecal BDNF produced a robust reduction in paw withdrawal thresholds that persisted throughout the experimental period (Figure 2B). In contrast, pretreatment with 10Panx significantly attenuated the development of BDNF-induced hyperalgesia during the initial phase following BDNF administration. Analysis of the area under the curve (AUC) confirmed a significant reduction in nociceptive sensitization in animals receiving 10Panx compared with BDNF-treated animals (Figure 2C). Interestingly, the antinociceptive effect of 10Panx was transient. Although mechanical thresholds remained significantly higher than those observed in BDNF-treated animals during the early phase of the experiment, this protective effect progressively diminished over time. As the inhibitory activity of 10Panx waned, animals gradually developed mechanical hypersensitivity, eventually reaching nociceptive thresholds comparable to those observed in the BDNF group (Figure 2D). Importantly, administration of 10Panx alone did not alter baseline mechanical sensitivity, indicating that the observed effects were not due to nonspecific changes in nociceptive processing but rather to inhibition of Panx1-dependent signaling pathways activated by BDNF. Together, these findings demonstrate that Panx1 channel activity contributes to the development of BDNF-induced mechanical hyperalgesia. The progressive reappearance of nociceptive sensitization following loss of 10Panx activity further suggests that sustained Panx1 channel function is required to maintain the persistent pain state induced by BDNF.

### 2.3. BDNF Promotes TrkB-Dependent Panx1 Phosphorylation and Channel Activation in Dorsal Horn Neurons

**Figure 3:**
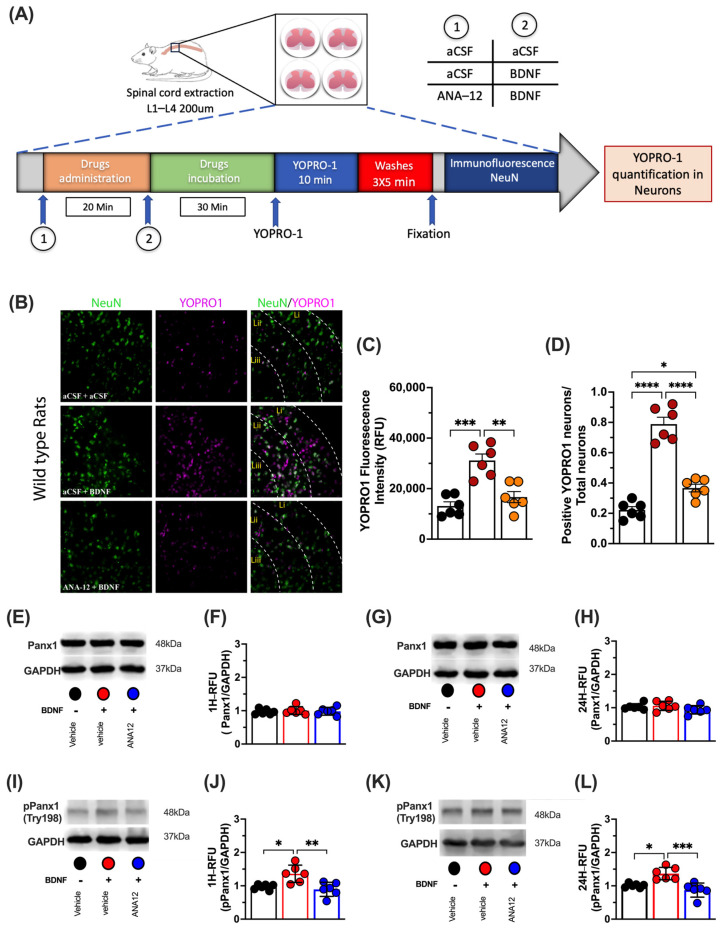
BDNF induces Panx1 activation in dorsal horn neurons through TrkB signaling. Ex vivo assessment of Panx1 channel activity and phosphorylation in spinal dorsal horn neurons following BDNF administration in wild-type rats. (**A**) Experimental scheme showing preparation of L1–L4 spinal cord slices, pretreatment and treatment administration, and assessment of YOPRO-1 uptake. (**B**) Representative confocal images of YOPRO-1 incorporation in NeuN-positive neurons located in laminae I-III of the spinal dorsal horn following treatment with vehicle, BDNF, or ANA12/BDNF. (**C**) Quantitative analysis of YOPRO-1 fluorescence intensity in NeuN-positive neurons. (**D**) Ratio of YOPRO-1-positive neurons to the total number of neurons analyzed in each condition. Representative Western blot images and densitometric analysis of total Panx1 expression at (**E**,**F**) 1 h and (**G**,**H**) 24 h after treatment. Representative Western blot images and densitometric analysis of phosphorylated Panx1 (Try198) at (**I**,**J**) 1 h and (**K**,**L**) 24 h after treatment. Data are expressed as mean ± SD (*n* = 6 animals per group). Statistical analysis was performed using Kruskal–Wallis test followed by Dunn’s multiple comparisons test. * *p* < 0.05, ** *p* < 0.01, *** *p* < 0.001, **** *p* < 0.0001.

To further investigate the role of BDNF-TrkB signaling in Panx1 activation during the development of pain, Panx1 channel activity was assessed functionally by YOPRO-1 uptake and biochemically by evaluating Panx1 phosphorylation using Western blot analysis. YOPRO-1 is a fluorescent dye with low membrane permeability under physiological conditions but readily enters cells following activation of large-pore membrane channels such as Panx1 [24,29]. YOPRO-1 incorporation was evaluated in lumbar spinal cord slices (L1–L4; 200 µm) obtained from wild-type rats (Figure 3A). Spinal cord slices treated with aCSF/aCSF exhibited minimal YOPRO-1 uptake (Figure 3B–D). In contrast, slices treated with aCSF/BDNF displayed a marked increase in YOPRO-1 incorporation in NeuN-positive neurons located within laminae I-III of the dorsal horn (Figure 3B–D). Quantitative analysis revealed that BDNF significantly increased YOPRO-1 fluorescence intensity compared with control slices (aCSF/aCSF: 13,131 ± 1642 vs. aCSF/BDNF: 31,151 ± 2563 RFU; Figure 3C). Consistent with these findings, approximately 80% of dorsal horn neurons were YOPRO-1 positive following BDNF treatment (Figure 3D). Importantly, pretreatment with the TrkB antagonist ANA12 significantly reduced YOPRO-1 incorporation (ANA12/BDNF: 16,657 ± 2194 RFU) and decreased the proportion of YOPRO-1-positive neurons to approximately 40% (Figure 3C,D).

To determine whether the increased YOPRO-1 uptake induced by BDNF was associated with changes in Panx1 expression or phosphorylation, lumbar spinal cord tissue was analyzed by Western blot at 1 h and 24 h after treatment. Total Panx1 protein levels remained unchanged at both time points following BDNF administration (Figure 3E–H). In contrast, BDNF significantly increased Panx1 phosphorylation (Try198) at both 1 h and 24 h, whereas pretreatment with ANA12 markedly reduced phospho-Panx1 levels (Figure 3I–L). Together, these findings demonstrate that BDNF activates Panx1 channels in dorsal horn neurons through a TrkB-dependent mechanism. While total Panx1 expression remained unchanged, BDNF increased both YOPRO-1 uptake and Panx1 phosphorylation, indicating that TrkB signaling promotes Panx1 activation through post-translational regulatory mechanisms rather than changes in channel expression.

### 2.4. TrkB Mediates Panx1 Activation via Src-Family Kinases

**Figure 4:**
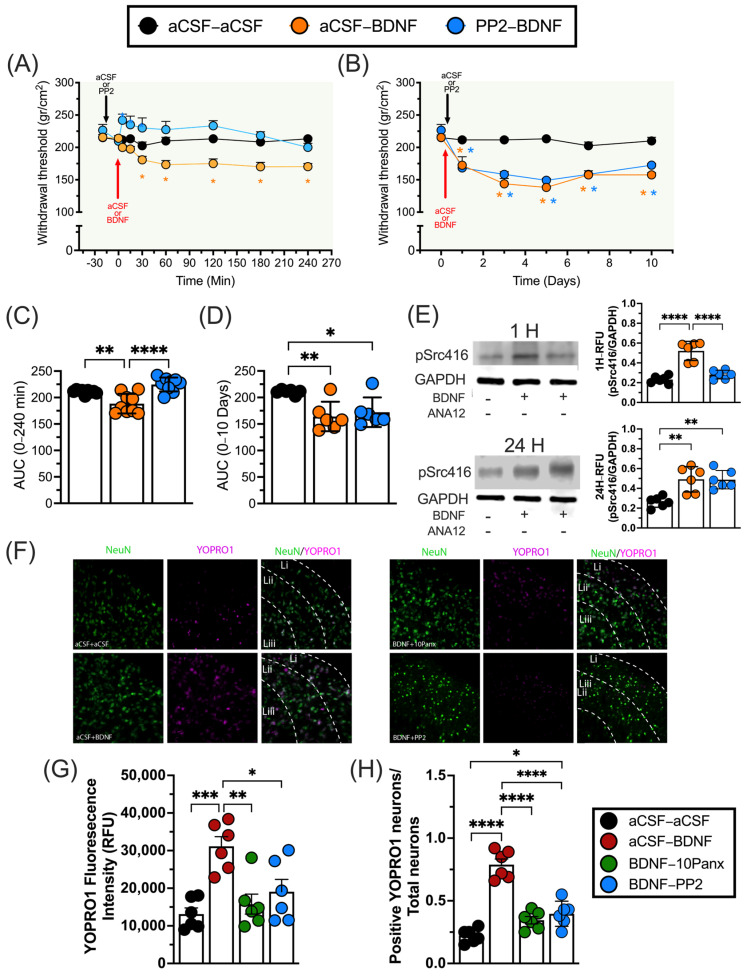
Src-family kinase mediates TrkB-dependent Panx1 activation. Effect of intrathecal (i.t.) administration of PP2 on early and late nociceptive responses induced by BDNF in wild-type rats. (**A**) Short-term (minutes) and (**B**) long-term (days) time-course effects of intrathecal administration of aCSF, BDNF (100 ng), and PP2/BDNF (10 µM/100 ng). Integrated responses are shown as the area under the curve (AUC) for (**C**) short-term (0–240 min) and (**D**) long-term (0–10 days) responses. (**E**) Representative Western blot images and densitometric analysis of phosphorylated Src (Tyr416) at 1 h and 24 h following treatment. (**F**) Representative confocal images of YOPRO-1 incorporation in NeuN-positive neurons located within laminae I-III of the spinal dorsal horn following treatment with vehicle, BDNF, 10Panx/BDNF, or PP2/BDNF. (**G**) Quantitative analysis of YOPRO-1 fluorescence intensity in NeuN-positive neurons. (**H**) Ratio of YOPRO-1-positive neurons to the total number of neurons analyzed in each condition. Data are expressed as mean ± SD (*n* = 6 animals per group). Changes over time within each group were analyzed using the Friedman test, followed by Dunn’s multiple comparisons test. Comparisons between groups at individual time points were performed using the Kruskal–Wallis test, followed by Dunn’s multiple comparisons test. The asterisks shown in the time-course panels indicate significant differences between experimental groups at the corresponding time points. AUC data were analyzed using the Kruskal–Wallis test, followed by Dunn’s multiple comparisons test. * *p* < 0.05, ** *p* < 0.01, *** *p* < 0.001, **** *p* < 0.0001.

Having established that BDNF promotes TrkB-dependent Panx1 activation, we next investigated whether Src-family kinases participate in the signaling pathway linking TrkB activation to Panx1 channel opening. Src family kinases are established downstream effectors of TrkB signaling and have been implicated in regulating Panx1 activity [20,22,23,24,30]. To evaluate the functional contribution of Src-family kinases signaling, wild-type rats received intrathecal administration of the Src-family kinases inhibitor PP2 prior to BDNF treatment (Figure 4A–D). Consistent with previous observations, BDNF administration produced a marked reduction in mechanical withdrawal thresholds during both the acute and prolonged phases of the nociceptive response. Pretreatment with PP2 significantly attenuated BDNF-induced hyperalgesia during the early phase of the response, as evidenced by the time-course analysis and area under the curve (AUC) measurements (Figure 4A,C). However, similar effects were observed with 10Panx, the antinociceptive effect of PP2 progressively diminished over time, resulting in only partial protection during the long-term phase (Figure 4B,D). To determine whether BDNF activates Src-family kinases signaling in the spinal cord, phosphorylated Src (Tyr416) levels were evaluated by Western blot. BDNF significantly increased Src416 phosphorylation at both 1 h and 24 h after treatment compared with vehicle-treated animals (Figure 4E). These findings indicate that activation of TrkB signaling is associated with sustained activation of Src-family kinases in the spinal dorsal horn.

We next investigated whether Src-family kinase activity contributes to Panx1 channel opening. Consistent with the results shown in Figure 3, BDNF markedly increased YOPRO-1 incorporation in neurons located within laminae I-III of the dorsal horn (Figure 4F). Pretreatment with either PP2 or the Panx1 inhibitor 10Panx significantly reduced YOPRO-1 uptake compared with BDNF-treated slices (Figure 4F–H). Quantitative analysis demonstrated a significant reduction in both total YOPRO-1 fluorescence intensity and the proportion of YOPRO-1-positive neurons following Src-family kinase inhibition (Figure 4G,H). Together, these findings demonstrate that Src kinase activity is required for BDNF-induced Panx1 activation and contributes to the development of nociceptive sensitization. The ability of PP2 to reduce both hyperalgesia and YOPRO-1 uptake, together with the observed increase in phospho-Src416 following BDNF treatment, supports a model in which Src-family kinases act as a critical signaling intermediate linking TrkB activation to Panx1 channel opening in dorsal horn neurons.

### 2.5. BDNF-Induced ATP Release Requires TrkB, Src-Family Kinases, and Panx1 Signaling

**Figure 5:**
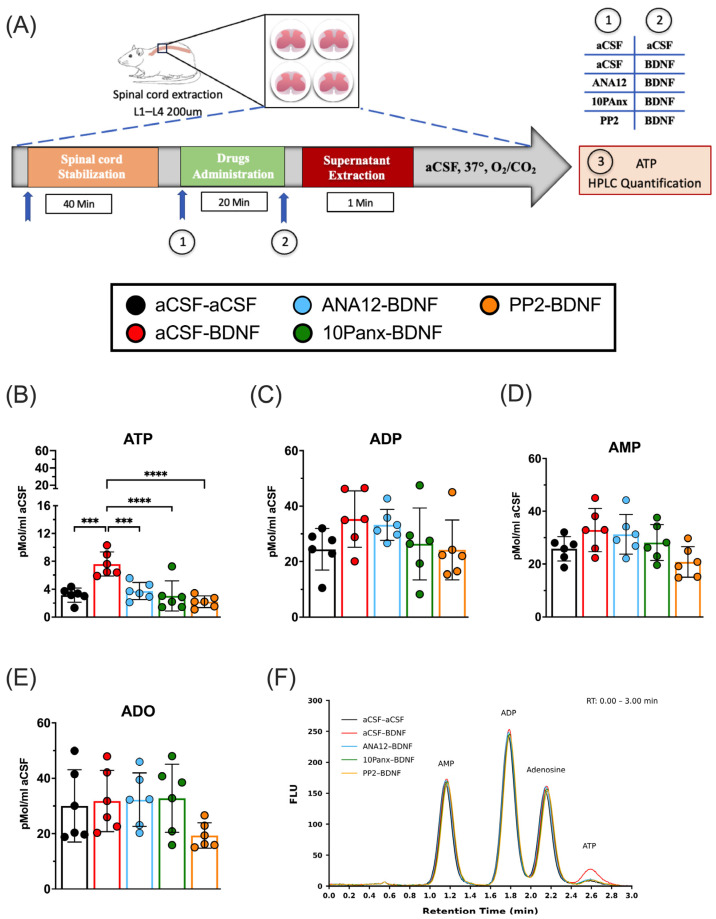
ATP release occurs downstream of TrkB-Src-Panx1 signaling in the spinal cord. (**A**) Experimental scheme illustrating ATP quantification in spinal cord slices following pharmacological modulation of the TrkB-Src-Panx1 pathway. HPLC quantification of extracellular (**B**) ATP, (**C**) ADP, (**D**) AMP, and (**E**) adenosine (ADO) in spinal cord supernatants following treatment with BDNF, ANA12/BDNF, 10Panx/BDNF, or PP2/BDNF. BDNF significantly increased extracellular ATP levels, whereas ADP, AMP, and adenosine concentrations remained unchanged. Inhibition of TrkB, Src-family kinases, or Panx1 prevented BDNF-induced ATP release. (**F**) Representative HPLC-UV chromatogram showing the separation and detection of ATP, ADP, AMP, and ADO. Data are expressed as mean ± SD (*n* = 6 animals per group). Statistical analysis was performed using the Kruskal–Wallis test followed by Dunn’s multiple comparisons test. *** *p* < 0.001, **** *p* < 0.0001.

Because Panx1 channels are major conduits for ATP release and ATP signaling has been strongly implicated in nociceptive sensitization, we next investigated whether activation of the BDNF-TrkB-Src-Panx1 pathway promotes ATP release from the spinal cord. ATP and its metabolites were quantified in the extracellular medium of lumbar spinal cord slices (L1–L4) using HPLC-UV following pharmacological treatments (Figure 5A). Treatment of spinal cord slices with BDNF significantly increased extracellular ATP levels compared with vehicle-treated controls (Figure 5B). Importantly, inhibition of TrkB signaling with ANA12 completely prevented the increase in ATP induced by BDNF. Similarly, blockade of Panx1 channels with 10Panx or inhibition of Src-family kinase activity with PP2 significantly reduced ATP release, restoring ATP concentrations toward control values (Figure 5B). To determine whether BDNF also altered extracellular ATP metabolism, the concentrations of ADP, AMP, and adenosine (ADO) were quantified. In contrast to ATP, no significant differences were observed in the extracellular levels of ADP, AMP, or ADO among the experimental groups (Figure 5C–E). These findings indicate that BDNF selectively promotes ATP accumulation in the extracellular space without significantly affecting the concentrations of its major metabolites under the experimental conditions examined.

Together, these findings demonstrate that BDNF promotes ATP release from spinal cord tissue through a signaling pathway that requires TrkB receptor activation, Src-family kinase activity, and Panx1 channel opening. The ability of ANA12, PP2, and 10Panx to suppress ATP release further supports a model in which Panx1 functions as a downstream effector of BDNF-TrkB signaling and contributes to ATP-dependent nociceptive sensitization.

## 3. Discussion

In the present study, we identify Panx1 as a previously unrecognized downstream effector of BDNF-TrkB signaling in dorsal horn neurons and establish its contribution to the initiation of BDNF-induced nociceptive sensitization. Behavioral experiments showed that a single intrathecal administration of BDNF induces mechanical hyperalgesia that persists for at least 10 days. Mechanistically, BDNF-induced sensitization was associated with TrkB-dependent activation of Src-family kinases, increased Panx1 phosphorylation and channel opening, and extracellular ATP accumulation. Pharmacological inhibition of Src-family kinases or Panx1 predominantly attenuated the early phase of the nociceptive response, supporting a role for this signaling pathway in initiating BDNF-induced sensitization. Collectively, these findings support a BDNF-TrkB–Src-family kinase–Panx1 signaling pathway linking neurotrophin signaling to ATP-dependent mechanisms during the development of prolonged nociceptive sensitization in the spinal dorsal horn.

BDNF is widely recognized as a critical regulator of neuronal plasticity and one of the most important mediators of central sensitization [12,31,32,33]. Increased BDNF expression has been reported in multiple chronic pain conditions, including neuropathic, inflammatory, and cancer-associated pain, where it contributes to long-lasting changes in synaptic transmission and neuronal excitability through activation of TrkB receptors [12,17,32,34,35]. Previous studies from our group demonstrated that a single intrathecal administration of BDNF is sufficient to induce persistent mechanical hypersensitivity lasting several days [15,16], a finding reinforced by independent studies that established BDNF as a critical mediator of nociceptive plasticity in the spinal dorsal horn [11]. Consistent with these observations, we found that a single intrathecal administration of BDNF produced mechanical hyperalgesia that persisted for at least 10 days, emphasizing the remarkable ability of this neurotrophin to induce long-lasting alterations in spinal nociceptive circuits.

An important finding of the present study is that the prolonged nociceptive state induced by BDNF remained dependent on TrkB receptor activation. Pharmacological inhibition of TrkB with ANA12 prevented both the early and late phases of BDNF-induced hyperalgesia, indicating that sustained nociceptive sensitization requires continued TrkB signaling. This observation is particularly intriguing considering that BDNF has a relatively short biological half-life in vivo [36]. Therefore, the persistence of hyperalgesia is unlikely to be explained solely by the presence of exogenously administered BDNF. Instead, our findings support the idea that BDNF-TrkB signaling initiates self-sustaining mechanisms that maintain nociceptive plasticity long after the initial stimulus has disappeared. Several mechanisms may contribute to the prolonged activation of TrkB signaling. Indeed, previous studies have shown that TrkB stimulation can autonomously induce delayed BDNF transcription in an activity-dependent manner [37] and increase the expression of multiple BDNF transcripts in neurons [38]. Furthermore, BDNF has been reported to act as an autocrine signaling molecule that promotes its own synthesis and release across diverse biological systems [39,40,41]. These positive feedback mechanisms may explain how a transient increase in BDNF initiates a persistent signaling state within dorsal horn neurons. In addition to BDNF-dependent positive feedback, several complementary mechanisms may contribute to the prolonged nociceptive effects observed following BDNF administration. BDNF-TrkB signaling can induce long-lasting changes in spinal synaptic efficacy by potentiating NMDA receptor signaling and spinal long-term potentiation [11,12,17,18]. Moreover, extracellular ATP may reinforce excitatory transmission by activating purinergic receptors and enhancing glutamatergic signaling within the dorsal horn [14,15,16,17,18,19,20,21]. ATP-dependent signaling may also recruit secondary neuron-glia communication through purinergic receptors expressed by microglia and astrocytes, potentially amplifying or prolonging nociceptive signaling. Thus, the persistence of BDNF-induced hyperalgesia may reflect the convergence of BDNF-dependent positive feedback, long-lasting synaptic plasticity, purinergic amplification, and neuron-glia communication rather than the prolonged action of a single molecular mechanism. These processes are not mutually exclusive and may form interconnected feed-forward loops that sustain spinal nociceptive plasticity after the initial exogenous BDNF stimulus has disappeared. Consistent with a sustained contribution of TrkB-dependent mechanisms, intrathecal administration of ANA12 has been shown to attenuate persistent pain behaviors across several experimental paradigms [35], supporting the notion that ongoing TrkB signaling contributes to the maintenance of BDNF-dependent nociceptive sensitization and may also participate in persistent pain conditions in which BDNF signaling is engaged.

The complexity of TrkB signaling may further contribute to the sustained effects observed following BDNF administration. Alternative splicing of TrkB generates multiple receptor isoforms, including the catalytically active full-length receptor (TrkB-FL) and truncated variants lacking intracellular kinase domains [42]. TrkB-FL and TrkB-T2 are predominantly expressed by neurons, whereas TrkB-T1 is highly expressed by astrocytes [43,44]. Because ANA12 binds to the extracellular domain of TrkB [45], its effects are not restricted to a single receptor isoform. Consequently, although our findings clearly demonstrate a critical role for TrkB signaling in BDNF-induced hyperalgesia, future studies will be required to determine the relative contribution of specific TrkB isoforms and different cellular populations to the maintenance of persistent pain.

A central finding of the present study is the identification of Panx1 as a downstream effector of BDNF-TrkB signaling during nociceptive sensitization. Although both BDNF signaling and the Panx1 channel have been implicated in chronic pain independently, a direct mechanistic connection between these pathways had not previously been established. Our results demonstrate that pharmacological inhibition of Panx1 with 10Panx significantly attenuated BDNF-induced mechanical hyperalgesia, indicating that Panx1 activity is required for the full expression of the nociceptive response induced by BDNF. Interestingly, the inhibitory effect of 10Panx was transient and progressively diminished over time. As the inhibitory peptide was cleared, animals gradually developed mechanical hypersensitivity, eventually reaching nociceptive thresholds comparable to those observed in BDNF-treated animals. This observation suggests that sustained BDNF-TrkB signaling persists upstream of Panx1 and continuously drives a nociceptive state. Consequently, Panx1 appears to function as an effector of the BDNF-TrkB pathway rather than as the initiating signal responsible for maintaining hyperalgesia.

The involvement of Panx1 in pain processing has been documented in several experimental models, including neuropathic pain, trigeminal hypersensitivity, migraine, and inflammatory pain [24,25,28,46]. Moreover, Panx1 is expressed in intrinsic neurons of the dorsal horn, where it co-localizes with the neuronal marker NeuN under both physiological and neuropathic conditions [24,25,27,47]. These observations are particularly relevant to the present study, in which Panx1 activation was evaluated in NeuN-positive neurons in laminae I-III of the dorsal horn. Importantly, the contribution of Panx1 to pain processing is not supported exclusively by pharmacological studies. Zhang and colleagues demonstrated that intrathecal administration of a Panx1-specific siRNA significantly attenuated neuropathic pain behaviors following spinal nerve ligation, providing direct evidence that molecular silencing of Panx1 reduces nociceptive sensitization [48]. Likewise, genetic ablation of Panx1 markedly reduces the development of chronic pain states in experimental models of neuropathic pain [49]. The convergence of pharmacological, molecular, and genetic evidence supports a causal role for Panx1 in pain-related plasticity and reinforces the interpretation that the effects observed in the present study are attributable to inhibition of Panx1 signaling. Additionally, we found that BDNF promoted Panx1 phosphorylation and channel opening. The increase in YOPRO-1 uptake observed following BDNF treatment was prevented by TrkB inhibition, indicating that Panx1 activation occurs downstream of BDNF-TrkB signaling. Importantly, we observed that total Panx1 expression remained unchanged at all time points examined, suggesting that BDNF regulates channel activity primarily through post-translational mechanisms rather than through changes in protein expression. The parallel increase in phospho-Panx1 levels and YOPRO-1 incorporation provides complementary biochemical and functional evidence supporting this conclusion. Our results do not demonstrate that this pathway universally mediates chronic pain but rather identify Panx1 as a downstream effector of BDNF signaling under the experimental conditions employed.

It is also noteworthy that alternative explanations for YOPRO-1 uptake are unlikely. Connexin 36, a neuronal gap junction protein highly expressed in dorsal horn neurons [50,51], is not permeable to YOPRO-1 under physiological conditions [52]. P2X7 receptors are well-established large-pore channels that can mediate YOPRO-1 uptake. Therefore, we cannot exclude the possibility that P2X7 receptors contribute to the dye uptake observed after BDNF treatment. Nevertheless, several observations support the involvement of Panx1 in this response. BDNF increased Panx1 phosphorylation without changing total Panx1 expression, 10Panx reduced YOPRO-1 uptake, and inhibition of TrkB or Src-family kinases attenuated the same functional readout. Moreover, pharmacological blockade of Panx1, TrkB, or Src-family kinases prevented BDNF-induced ATP accumulation. Because extracellular ATP can activate P2X7 receptors, it is possible that Panx1-mediated ATP release and P2X7 activation operate as coupled or sequential mechanisms amplifying YOPRO-1 uptake. Thus, our data support Panx1 activation downstream of BDNF-TrkB signaling but do not exclude P2X7 as a parallel or downstream contributor to the YO-PRO-1 signal. Future studies using selective P2X7 antagonists or genetic approaches will be required to determine the relative contribution of Panx1 and P2X7 receptors in this model.

The molecular mechanisms linking TrkB activation to Panx1 channel opening have never been explored before. TrkB receptors activate multiple intracellular signaling pathways, including members of the Src family kinases, which play important roles in neuronal excitability, synaptic plasticity, and nociceptive transmission [19,31]. Previous studies have demonstrated that Src family kinases can directly regulate Panx1 channel activity through phosphorylation-dependent mechanisms [22,23,24,27]. However, although Src-family kinase-dependent regulation of Panx1 has previously been demonstrated in neuropathic pain models, whether BDNF-TrkB signaling functions as the upstream molecular trigger engaging this pathway during nociceptive sensitization has not been previously investigated. Our findings provide several lines of evidence supporting a role for Src kinase as a critical intermediary between TrkB activation and Panx1 channel opening. First, intrathecal administration of BDNF increased phosphorylation of Src at Tyr416, a well-established marker of Src activation [22,23,53]. Second, pharmacological inhibition of Src-family kinases with PP2 significantly attenuated BDNF-induced mechanical hyperalgesia during the early phase of the nociceptive response. Third, PP2 reduced BDNF-induced YOPRO-1 uptake in dorsal horn neurons, indicating that Src-family kinases activity is required for Panx1 channel opening downstream of TrkB activation. Together, these findings support the existence of a TrkB-Src-Panx1 signaling axis that contributes to the initiation of nociceptive sensitization. Interestingly, the effects of PP2 closely resembled those observed following Panx1 inhibition with 10Panx. In both cases, inhibition significantly reduced the early nociceptive response but failed to permanently prevent the development of long-term hyperalgesia. Once the inhibitory effects of PP2 or 10Panx diminished, mechanical hypersensitivity progressively re-emerged. These observations suggest that Src-family kinases and Panx1 act downstream of a persistent upstream TrkB-dependent signaling process. Thus, while Src-family kinase activation is required for the acute expression of BDNF-induced nociceptive signaling, continued TrkB activation appears capable of re-establishing the pathway once pharmacological inhibition is removed.

Our results also extend previous observations from neuropathic pain models. We recently demonstrated that Src-mediated Panx1 activation contributes to spontaneous mechanical hypersensitivity following peripheral nerve injury and that inhibition of Src-family kinases reduces both Panx1 channel activity and pain-related behaviors [24]. The present study substantially extends these findings by identifying BDNF-TrkB signaling as the upstream molecular trigger that engages the previously described Src-family kinase–Panx1 pathway. In addition, we demonstrate that activation of this signaling cascade promotes ATP release from spinal neurons, thereby providing a mechanistic framework linking neurotrophin signaling to purinergic modulation of nociceptive sensitization. Consequently, Src appears to occupy a central position at the intersection of neurotrophin signaling and pannexin-mediated purinergic communication during nociceptive sensitization. An intriguing observation was the increase in phosphorylated Src416 detected 24 h after BDNF administration. While ANA12 effectively prevented the development of long-term mechanical hyperalgesia, elevated pSrc416 levels were still detected at later time points. Although the precise mechanism responsible for this delayed Src activation remains unclear, several possibilities may be considered. One potential explanation is that ATP released through activated Panx1 channels promotes glutamate release from primary afferent terminals and enhances NMDA receptor signaling within dorsal horn neurons [54,55,56]. Since NMDA receptor activation can itself stimulate Src416 phosphorylation [24], ATP-dependent glutamatergic signaling may contribute to a secondary wave of Src activation distinct from the initial TrkB-dependent response. Alternatively, ATP released from neuronal Panx1 channels may engage purinergic signaling pathways that promote the release of pro-nociceptive mediators capable of sustaining Src activation over time [25,26,55]. Although these possibilities remain speculative, they suggest that Src-family kinases may participate not only in the initiation but also in the maintenance of persistent nociceptive signaling. Taken together, these findings identify Src-family kinases as a critical molecular bridge linking TrkB activation to Panx1 channel opening. By coupling neurotrophin signaling to pannexin-mediated ATP release, Src appears to play a central role in propagating BDNF-induced nociceptive sensitization and represents a potential therapeutic target for disrupting pathological pain signaling.

A major consequence of Panx1 channel activation is the release of ATP, a signaling molecule that plays a central role in nociceptive transmission and central sensitization [57]. Accordingly, one of the most important findings of the present study is that activation of the BDNF-TrkB-Src-Panx1 pathway culminates in a significant increase in extracellular ATP levels within the spinal cord. Importantly, ATP release was prevented by inhibiting TrkB, Src-family kinases, or Panx1, providing functional evidence that ATP release occurs downstream of the identified signaling cascade. Although vesicular exocytosis represents an established mechanism of ATP release in neuronal and glial cells, whether BDNF-TrkB signaling directly promotes vesicular ATP release in spinal cord neurons remains unclear. Vesicular ATP release was not directly examined in the present study; therefore, its potential contribution to extracellular ATP accumulation cannot be excluded. Nevertheless, the marked reduction in BDNF-induced ATP accumulation following 10Panx treatment provides functional evidence supporting a contribution of Panx1 to this response. These findings should not be interpreted as establishing Panx1 as the exclusive ATP-release pathway, and future studies directly targeting vesicular ATP loading or exocytosis will be required to determine the relative contributions of vesicular and channel-mediated ATP-release mechanisms. ATP is widely recognized as a neurotransmitter and a danger-associated signaling molecule that modulates neuronal excitability, synaptic transmission, and neuroimmune communication [49,58,59]. Following its release into the extracellular space, ATP can activate a variety of purinergic receptors expressed throughout the nociceptive pathway, including P2X and P2Y receptor families located on neurons, microglia, astrocytes, and primary afferent terminals [26,60,61]. Through these actions, ATP contributes to the amplification and maintenance of pain signaling under both physiological and pathological conditions. Interestingly, while BDNF increased extracellular ATP, no significant changes were observed in ADP, AMP, or adenosine levels, suggesting that BDNF primarily promotes ATP accumulation in the extracellular space without substantially altering ATP metabolism under these experimental conditions. The selective increase in ATP, together with its sensitivity to 10Panx, further supports the contribution of Panx1 channel activity to extracellular ATP accumulation following BDNF stimulation. The identification of ATP as a downstream product of BDNF-TrkB-Src-Panx1 signaling may also provide insight into the persistence of BDNF-induced hyperalgesia. A single intrathecal administration of BDNF induced mechanical hypersensitivity that persisted for at least 10 days, despite the neurotrophin’s relatively short half-life [62]. ATP represents an attractive candidate mediator capable of sustaining nociceptive signaling beyond the initial period of TrkB activation. In support of this possibility, previous studies have shown that intrathecal administration of ATP or the stable P2X receptor agonist BzATP produces prolonged nociceptive sensitization that can persist for days or even weeks [59,63]. In contrast, intrathecal NMDA administration generally induces shorter-lasting nociceptive responses [64,65,66,67]. Thus, ATP released through Panx1 channels may represent a key signaling intermediate linking transient BDNF exposure to long-lasting nociceptive plasticity. Collectively, these findings identify ATP release as a major functional outcome of BDNF-dependent Panx1 activation and suggest that purinergic signaling represents an important mechanism through which BDNF promotes sustained nociceptive sensitization in the present experimental model. Because BDNF signaling is activated in multiple persistent pain conditions, this pathway may also contribute to pathological pain states and warrants further investigation in disease-relevant models.

Although the present study was designed to investigate neuronal mechanisms downstream of BDNF-TrkB signaling, ATP released through Panx1 channels may have broader implications for neuron-glia communication in the spinal cord. Following its release into the extracellular space, ATP can activate multiple purinergic receptor subtypes expressed by neurons, microglia, and astrocytes, thereby modulating nociceptive processing at several levels of the pain pathway [26,60,61,68]. Importantly, our findings demonstrate that BDNF-induced Panx1 activation occurs in NeuN-positive neurons located within laminae I-III of the dorsal horn. Furthermore, BDNF increased both Panx1 phosphorylation and YOPRO-1 uptake in these neurons, indicating a neuronal source of ATP release. Thus, the primary mechanism established in the present study is a neuronal BDNF-TrkB-Src-Panx1 signaling pathway that culminates in extracellular ATP accumulation. While direct activation of microglia or astrocytes was not evaluated, ATP released via neuronal Panx1 channels may serve as a critical signaling intermediate, recruiting downstream neuroimmune mechanisms that contribute to persistent pain. This interpretation may help integrate the present findings with previous studies reporting important roles for both BDNF and glial signaling in chronic pain. BDNF has been shown to modulate microglial and astrocytic function in several pathological conditions, while ATP represents one of the most effective signaling molecules linking neuronal activity to glial responses [69,70]. Therefore, neuronal ATP release downstream of TrkB-Src-Panx1 signaling may constitute a mechanistic bridge between neurotrophin-dependent neuronal plasticity and the broader neuroimmune processes that characterize central sensitization. Future studies should determine the extent to which microglia and astrocytes contribute to the downstream consequences of ATP release following BDNF-induced Panx1 activation.

An important consideration of the present study is that all experiments were performed in male rats. This experimental design was selected to maintain consistency with previous studies investigating BDNF-induced nociceptive sensitization and to minimize variability associated with hormonal fluctuations known to influence pain processing. More importantly, accumulating evidence indicates that BDNF-dependent pain mechanisms exhibit marked sexual dimorphism, suggesting that the contribution of BDNF signaling to chronic pain may differ substantially between males and females. Studies in rodents have demonstrated that microglia-dependent pain mechanisms are more prominent in males than in females [71,72]. In male animals, activation of spinal microglia promotes the release of mediators, including BDNF, which contribute to neuronal disinhibition and central sensitization. In contrast, females appear to rely more heavily on alternative immune pathways involving adaptive immune cells, suggesting that distinct neuroimmune mechanisms may underlie chronic pain in the two sexes [71]. Importantly, sex-dependent differences in BDNF signaling have also been observed beyond experimental pain models. Several studies have reported differences in BDNF expression, TrkB signaling, and neuroimmune interactions between males and females under physiological and pathological conditions [72,73]. Moreover, recent translational studies using human spinal cord tissue have demonstrated that BDNF-induced signaling pathways associated with pain sensitization differ between males and females, consistent with observations previously reported in rodent models [74]. Therefore, future studies should determine whether BDNF-induced Panx1 activation and ATP release occur similarly in females or whether alternative signaling pathways predominate. These investigations will be essential for defining the generalizability of the TrkB-Src-Panx1 pathway and for understanding potential sex-specific therapeutic opportunities targeting Panx1 signaling.

While the present findings provide strong evidence for a role of the BDNF-TrkB-Src-Panx1 pathway in sustained nociceptive sensitization, several questions remain regarding the cellular and molecular mechanisms underlying its long-term regulation. An additional limitation is that the experimental model used in the present study relies on exogenous intrathecal BDNF administration rather than on a disease or nerve injury model. Therefore, our conclusions should be interpreted as defining signaling mechanisms downstream of BDNF activation rather than establishing a universal mechanism underlying chronic pain. First, the mechanistic conclusions of this study are based primarily on pharmacological interventions, including ANA12, PP2, and 10Panx. Although these compounds have been extensively validated and are widely used to investigate TrkB, Src family kinases, and Panx1 signaling [24,25,28,75], the possibility of off-target effects cannot be completely excluded. To minimize this concern, we employed multiple pharmacological approaches targeting distinct components of the proposed signaling cascade and observed highly consistent effects across behavioral, biochemical, and functional experiments. Importantly, the interpretation that Panx1 contributes to nociceptive sensitization is supported by a broader body of evidence extending beyond pharmacological inhibition. Previous studies have demonstrated that intrathecal administration of Panx1-specific siRNA significantly attenuates neuropathic pain behaviors following peripheral nerve injury [48], and genetic deletion of Panx1 markedly reduces the development of chronic pain states in experimental models of neuropathic pain [46]. The convergence of pharmacological, molecular, and genetic evidence strongly supports a causal role for Panx1 in pain-related plasticity and reinforces the interpretation that the effects observed in the present study are attributable to inhibition of Panx1 signaling. A second limitation is that the present study was specifically designed to investigate neuronal mechanisms downstream of BDNF-TrkB signaling. Consequently, although we demonstrate Panx1 activation and ATP release in dorsal horn neurons, we did not directly evaluate the contribution of microglia or astrocytes to the downstream consequences of ATP signaling. Given the established role of purinergic signaling in neuron-glia communication during central sensitization, future studies should determine whether glial activation contributes to the maintenance of the BDNF-TrkB-Src-Panx1 pathway. Finally, future studies employing cell-specific genetic approaches, including conditional Panx1 deletion or targeted silencing strategies, will be valuable for defining the relative contribution of neuronal and non-neuronal Panx1 signaling to chronic pain. Such approaches may also help clarify the temporal relationship between TrkB activation, ATP release, neuroimmune signaling, and the transition from acute nociceptive responses to persistent pain states.

## 4. Materials and Methods

### 4.1. Animals

Adult male Sprague-Dawley rats (180–200 g) were obtained from the Animal Facility of the Faculty of Chemical and Pharmaceutical Sciences, University of Chile. Animals were housed under controlled temperature (24 °C) and a 12 h light/dark cycle, with food and water available ad libitum. Only male rats were used in this study to minimize biological variability associated with estrous cycle-dependent fluctuations in pain sensitivity and BDNF signaling. In addition, previous studies have demonstrated that BDNF-induced pain sensitization exhibits marked sex-dependent differences, with stronger pronociceptive effects reported in males than in females [35,73,76]. Therefore, the use of male animals allowed direct comparison with previously established BDNF-induced pain models and facilitated mechanistic evaluation of the BDNF-TrkB-Panx1 signaling pathway. All experimental procedures were approved by the Ethics Committee of the University of Santiago de Chile (Approval No. 274/2023) and conducted in accordance with the ARRIVE guidelines and the Guide for the Care and Use of Laboratory Animals [77,78].

### 4.2. Pain Model Induced by Intrathecal Administration of BDNF

Sustained mechanical hyperalgesia was induced in wild-type male Sprague-Dawley rats by a single intrathecal (i.t.) administration of recombinant brain-derived neurotrophic factor (BDNF, Cat. No. 248-BDB, Tocris Bioscience, Bristol, UK), as previously described [16]. Briefly, rats were anesthetized with 3% isoflurane and received an intrathecal injection of BDNF (100 ng/10 µL; approximately 0.38 µM) into the L5-L6 intervertebral space using a 25 µL Hamilton syringe fitted with a 25-G needle. Control animals received an equivalent volume of artificial cerebrospinal fluid (aCSF). This experimental model has been previously shown to induce long-lasting spinal sensitization and persistent mechanical hyperalgesia through activation of BDNF-TrkB signaling pathways [11,15,16]. For all subsequent behavioral, biochemical, and imaging experiments, wild-type rats received intrathecal administration of vehicle or BDNF, as described in the experimental protocols below.

### 4.3. Drugs and Treatments

Wild-type rats received intrathecal (i.t.) administration of pharmacological inhibitors in a final volume of 10 µL, 20 min before BDNF administration. Animals received vehicle (artificial cerebrospinal fluid, aCSF), the selective TrkB antagonist ANA12 (100 nM) [79], the Panx1 inhibitory peptide 10Panx (300 µM) [24,25,28], or the Src-family kinase inhibitor PP2 (10 µM) [24,80]. The concentration of ANA12 was selected based on previously published evidence demonstrating effective inhibition of TrkB [79]. Animals subsequently received a second intrathecal injection of either vehicle (aCSF) or BDNF (100 ng in 10 µL). Experimental groups and treatment schedules are summarized in Table 1. ANA12 (Cat. No. SML0209, Sigma-Aldrich, St. Louis, MO, USA), PP2 (Cat. No. 1407, Tocris Bioscience, Bristol, UK), and 10Panx (GenScript, Piscataway, NJ, USA) were prepared according to the manufacturers’ instructions. ANA12 and 10Panx were dissolved in aCSF, whereas PP2 was initially dissolved in dimethyl sulfoxide (DMSO) to prepare a stock solution and subsequently diluted in aCSF to the final working concentration. Control animals received the corresponding vehicle solution.

### 4.4. Behavioral Assessment of Nociception

Mechanical hyperalgesia was assessed using the hind paw withdrawal pressure test (Randall–Selitto test), as previously described [81]. Mechanical pressure was applied at a constant rate to the mid-plantar surface of the hind paw using an analgesiometer (Ugo Basile, Gemonio, Varese, Italy). The nociceptive threshold was defined as the force (g/cm^2^) required to elicit paw withdrawal or vocalization. To ensure measurement reliability, each determination was repeated 2–3 times until two consecutive values differed by less than 15%, and the mean of these values was considered the withdrawal threshold. A cut-off value of 480 g/cm^2^ was established to prevent tissue damage.

Mechanical sensitivity was evaluated in all experimental groups (*n* = 6 per group). Animals were habituated to the testing environment and procedure on at least three separate occasions before the experimental day. Baseline nociceptive thresholds were obtained before drug administration. Animals subsequently received the pharmacological treatments described in Table 1, followed by intrathecal administration of vehicle or BDNF 20 min later. Mechanical thresholds were measured at 5, 15, 30, 60, 120, 180, and 240 min, and at 1, 3, 5, 7, and 10 days after BDNF administration. All behavioral experiments and data analyses were performed by investigators blinded to the treatment groups to minimize experimental bias.

### 4.5. Spinal Cord Extraction and Sectioning

Animals were deeply anesthetized with isoflurane (5%) and transcardially perfused with phosphate-buffered saline (PBS). The lumbar spinal cord (L1–L4 segments), including the dorsal horn region, was rapidly dissected and embedded in 4% low-gelling-temperature agarose (Agarose Type IX-A, Sigma-Aldrich, St. Louis, MO, USA). Transverse spinal cord slices (200 µm thick) were prepared using a vibratome (7000smz-2, Campden Instruments, Loughborough, UK) in ice-cold oxygenated artificial cerebrospinal fluid (aCSF) containing (in mM): 125 NaCl, 2.5 KCl, 25 glucose, 25 NaHCO_3_, 1.25 NaH_2_PO_4_, 2 CaCl_2_, and 1 MgCl_2_. Slices were continuously oxygenated with 95% O_2_/5% CO_2_ and maintained in aCSF at 37 °C for 40 min before experimental use. Lumbar spinal cord slices from L1–L4 were subsequently used for YOPRO-1 uptake assays, ATP release measurements, and biochemical analyses.

### 4.6. Dye Uptake Assay (YOPRO-1) and Immunofluorescence

Lumbar spinal cord slices (L1–L4; 200 µm) were prepared as described above (Section 4.5). YOPRO-1 uptake was used as an indirect measure of Panx1 channel opening, as previously reported [24,82,83]. Following the recovery period, slices were transferred to oxygenated aCSF and subjected to the pharmacological treatments described in Table 2. Briefly, slices received the compounds indicated in the first column of Table 2, followed 20 min later by the treatments indicated in the second column. 20 min after the second treatment, slices were incubated with 5 µM YOPRO-1 iodide (YOPRO™-1 Iodide, Thermo Fisher Scientific, Waltham, MA, USA) for 10 min at room temperature in oxygenated aCSF protected from light. Slices were subsequently washed with PBS and fixed in 4% paraformaldehyde for 1 h at room temperature.

For neuronal identification, slices were permeabilized and blocked in PBS containing 5% normal horse serum and 0.03% Triton X-100, followed by overnight incubation at 4 °C with anti-NeuN antibody (1:1000; E4M5P, Cat. No. 94403, Cell Signaling Technology, Danvers, MA, USA). After washing, slices were incubated with Alexa Fluor 647-conjugated anti-mouse secondary antibody (1:1000; Cat. No. A-21422, Thermo Fisher Scientific, Waltham, MA, USA) for 1 h at room temperature. Samples were mounted using Fluoromount-G (eBioscience™, Thermo Fisher Scientific, Waltham, MA, USA) and imaged using a Zeiss LSM 800 confocal microscope equipped with a GaAsP detector and a 63× oil-immersion objective (Carl Zeiss, Oberkochen, Germany).

### 4.7. Quantification of Dye Uptake

YO-PRO-1 uptake was quantified in NeuN-positive neurons located within laminae I–III of the dorsal horn according to the Rexed classification. Neurons were identified based on NeuN immunoreactivity and a minimum nuclear area of 25 µm^2^. Image analysis was performed using Fiji/ImageJ software (version 1.54j, National Institutes of Health, Bethesda, MD, USA). Regions of interest (ROIs) corresponding to NeuN-positive neurons were automatically selected using the Segmentation plugin. The resulting ROIs were subsequently transferred to the YOPRO-1 channel, and the mean gray value of YOPRO-1 fluorescence was measured within each neuronal ROI. Background fluorescence was determined from a cell-free region within the same microscopic field and subtracted from the mean gray value obtained for each neuronal ROI. The resulting background-corrected fluorescence values were expressed as relative fluorescence units (RFU) and used for quantitative analysis. Each biological replicate (n) consisted of three independent confocal images obtained from the dorsal horn and included the analysis of at least 30 neurons.

### 4.8. Western Blot Analysis

Western blot analyses were performed using tissue obtained from the lumbar spinal cord (L1–L4). Following tissue collection, the dorsal portion of the spinal cord containing both dorsal horns was rapidly isolated and homogenized in cell lysis buffer (Invitrogen, Carlsbad, CA, USA) supplemented with protease inhibitor cocktail (Sigma-Aldrich, St. Louis, MO, USA) and phosphatase inhibitor cocktail 2 (Sigma-Aldrich, St. Louis, MO, USA). Samples were centrifuged at 15,000 rpm for 30 min at 4 °C, and protein concentrations were determined using the bicinchoninic acid (BCA) assay (Thermo Fisher Scientific, Waltham, MA, USA). Equal amounts of protein (20 µg) obtained from six independent animals per experimental group were separated by SDS-PAGE and transferred onto nitrocellulose membranes. Membranes were blocked with 5% bovine serum albumin (BSA) in PBS containing 0.05% Tween-20 and incubated overnight at 4 °C with the following primary antibodies: anti-Panx1 (1:1000, Cat. No. AB9886, Millipore, Burlington, MA, USA), anti phospho-Panx1 (Try198) (1:500, Cat. No. ABN1681, Millipore, Burlington, MA, USA), anti-phospho-Src (Tyr416) (1:500, Cat. No. 2101, Cell Signaling Technology, Danvers, MA, USA), and anti-GAPDH (1:10,000, Cat. No. AB2302, Sigma-Aldrich, St. Louis, MO, USA). After washing, membranes were incubated with horseradish peroxidase-conjugated secondary antibodies (1:10,000; Thermo Fisher Scientific, Waltham, MA, USA) for 1 h at room temperature. Immunoreactive bands were visualized using enhanced chemiluminescence substrate (Clarity Western ECL Substrate, Cat. No. 1705061, Bio-Rad, Hercules, CA, USA) and imaged using Image Studio software 6.2 (LI-COR Biosciences, Lincoln, NE, USA). Band intensities were quantified by densitometric analysis using Image Studio software. For each sample, the intensities of the Panx1, phospho-Panx1, and phospho-Src (Tyr416) bands were normalized to the corresponding GAPDH signal. Data were subsequently normalized to the control group mean and expressed as relative protein expression.

### 4.9. ATP Release Quantification

ATP release was quantified from lumbar spinal cord slices (L1–L4) using high-performance liquid chromatography coupled to ultraviolet detection (HPLC-UV). Spinal cord slices were prepared as described above and allowed to recover for 40 min at 37 °C in oxygenated artificial cerebrospinal fluid (aCSF; 95% O_2_/5% CO_2_). The incubation solution contained (in mM): 120 NaCl, 3.1 KCl, 1 MgCl_2_, 1 CaCl_2_, 1.25 NaH_2_PO, pH 7.4. Following recovery, slices were transferred to individual wells containing oxygenated aCSF and subjected to the pharmacological treatments described in Table 2. Briefly, slices received the compounds indicated in the first column of Table 2, followed 20 min later by the treatments indicated in the second column. 15 min after the second treatment, 200 µL of extracellular medium was collected and immediately processed for nucleotide stabilization using citrate-phosphate buffer and perchloric acid. ATP and its metabolites, adenosine diphosphate (ADP), adenosine monophosphate (AMP), and adenosine (ADO), were quantified by HPLC-UV at 260 nm using a reverse-phase C18 column (4.6 × 150 mm, 5 µm) protected by a guard column (4.0 × 20 mm, 5 µm). The mobile phase consisted of 10 mM KH_2_PO_4_ buffer (pH 7.0) containing 5% acetonitrile at a flow rate of 1 mL/min. Chromatographic data were acquired using ChromGate software (version 3.3.3, Knauer, Berlin, Germany). Quantification was performed using external ATP standards (1 pM-50 µM), and ATP concentrations were calculated from calibration curves generated for each experimental series. Each biological replicate consisted of spinal cord slices from a single animal.

### 4.10. Statistical Analysis

Data are presented as mean ± SD. Statistical analyses were performed using GraphPad Prism 11 (GraphPad Software, San Diego, CA, USA). Experimental group sizes were determined according to the principles of Replacement, Reduction, and Refinement (3Rs), while maintaining sufficient statistical power to detect biologically meaningful effects. Group sizes of six animals per condition are consistent with those used in our previous studies investigating BDNF-, NMDA-, and Panx1-dependent pain mechanisms [15,24,28,75], as well as with sample sizes commonly reported in the experimental pain literature [48,84,85].

Due to the limited sample sizes and the assumption that the data distributions were not normally distributed, non-parametric statistical methods were used throughout the study. Behavioral time-course data were analyzed in two complementary ways. Changes over time within each experimental group were analyzed using the Friedman test, followed by Dunn’s multiple comparisons test. Comparisons between experimental groups at each individual time point were performed using the Kruskal–Wallis test followed by Dunn’s multiple-comparisons test. Area under the curve (AUC) data were analyzed using the Kruskal–Wallis test, followed by Dunn’s multiple comparisons test. Comparisons among independent experimental groups were performed using the Kruskal–Wallis test followed by Dunn’s multiple-comparisons post hoc test. For YOPRO-1 uptake, Western blot, and ATP release experiments, comparisons among multiple groups were also analyzed using the Kruskal–Wallis test followed by Dunn’s multiple-comparisons test. Statistical significance was accepted at *p* < 0.05.

## 5. Conclusions

**Figure 6:**
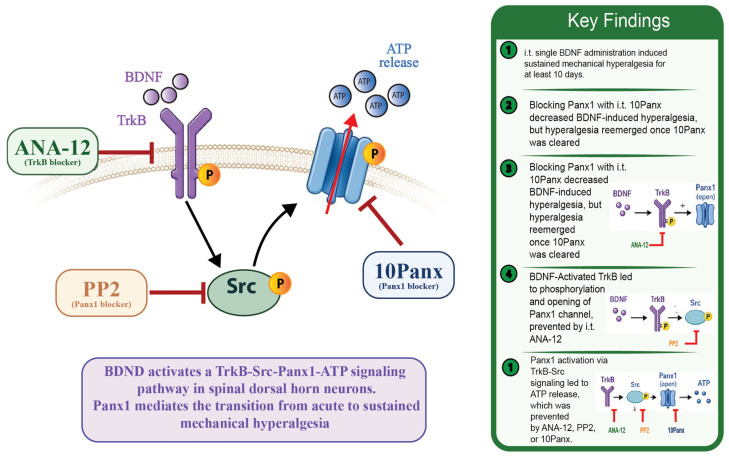
BDNF drives sustained mechanical hyperalgesia through a TrkB-Src-Panx1-ATP signaling pathway. Intrathecal administration of BDNF induces long-lasting mechanical hyperalgesia by activating TrkB receptors in dorsal horn neurons of the spinal cord. TrkB activation stimulates Src kinase activity, promoting Panx1 phosphorylation and channel opening, resulting in ATP release and contributing to central sensitization. Pharmacological inhibition of TrkB (ANA12), Src kinase (PP2), or Panx1 (10Panx) disrupts this signaling cascade and attenuates hyperalgesia. These findings identify the TrkB-Src-Panx1-ATP axis as a key mechanism underlying persistent pain sensitization and highlight Panx1 as a potential therapeutic target for chronic pain.

The present study identifies an unrecognized signaling pathway linking BDNF-TrkB activation to Panx1 channel opening in spinal dorsal horn neurons. We demonstrate that BDNF induces sustained nociceptive sensitization through a mechanism involving Src activation, Panx1 phosphorylation, Panx1 channel opening, and ATP release. These findings position Panx1 as a critical downstream effector of BDNF-TrkB signaling and expand our understanding of how neurotrophin-dependent signaling contributes to the development of persistent pain states. Importantly, our results suggest that ATP release represents a key functional consequence of BDNF-induced Panx1 activation (Figure 6). Given the established roles of ATP in neuronal excitability, purinergic signaling, and neuron-glia communication, the TrkB-Src-Panx1 pathway identified here may provide a mechanistic framework that links transient neurotrophin signaling to long-lasting nociceptive plasticity. We propose that ATP released through neuronal Panx1 channels serves as a critical signaling intermediate that sustains and amplifies pain-related signaling networks within the spinal cord. From a translational perspective, these findings identify Panx1 as an attractive therapeutic target positioned downstream of BDNF-TrkB signaling. While direct inhibition of neurotrophin signaling may interfere with essential physiological functions of BDNF, targeting Panx1 could provide a more selective strategy to interrupt pathological signaling pathways associated with chronic pain. Thus, modulation of Panx1 activity, either directly or through upstream regulators such as Src-family kinases, may represent a promising approach for developing novel therapies to prevent or reverse persistent nociceptive sensitization. Collectively, we propose a model in which BDNF activates TrkB receptors in dorsal horn neurons, leading to Src-dependent Panx1 activation and subsequent ATP release. Through this mechanism, Panx1 serves as a critical molecular link between neurotrophin signaling and purinergic pathways involved in chronic pain, thereby contributing to the transition from acute nociceptive responses to sustained pain sensitization.

## Figures and Tables

**Table 1:** In vivo drug administration.

Compound	
First Administration (Time 0)	Second Administration (20 min)
aCSF	aCSF
aCSF	BDNF (100 ng/10 µL)
ANA12 (100 nM)	BDNF (100 ng/10 µL)
PP2 (10 µM)	BDNF (100 ng/10 µL)
10Panx (300 µM)	BDNF (100 ng/10 µL)

**Table 2:** Spinal cord section treatments.

Compound	
First Administration	Second Administration (20 min)
aCSF (10 μL)	aCSF (10 μL)
aCSF (10 μL)	BDNF (0.38 μM)
ANA12 (100 nM)	BDNF (0.38 μM)
PP2 (10 µM)	BDNF (0.38 μM)
10Panx (300 μM)	BDNF (0.38 μM)

## Data Availability

Data are available by contacting the corresponding author.

## Abbreviations

The following abbreviations are used in this manuscript:

BDNF	Brain-derived neurotrophic factor
TrkB receptor	Tropomyosin receptor kinase B
Panx1	Pannexin 1 channel
NMDAR	N-methyl-D-aspartate receptor
